# Timing matters! Academic assessment changes throughout the day

**DOI:** 10.3389/fpsyg.2025.1605041

**Published:** 2025-07-24

**Authors:** Carmelo M. Vicario, Michael A. Nitsche, Chiara Lucifora, Pietro Perconti, Mohammad A. Salehinejad, Francesco Tomaiuolo, Simona Massimino, Alessio Avenanti, Massimo Mucciardi

**Affiliations:** ^1^Dipartimento di Scienze Cognitive, Psicologiche, Pedagogiche e degli studi culturali, Università di Messina, Messina, Italy; ^2^Department of Psychology and Neurosciences, Leibniz Research Centre for Working Environment and Human Factors (IfADo), Dortmund, Germany; ^3^Dipartimento di Filosofia e Comunicazione, Università di Bologna, Bologna, Italy; ^4^School of Cognitive Sciences, Institute for Research in Fundamental Sciences (IPM), Tehran, Iran; ^5^Department of Child and Adolescent Psychiatry, Psychosomatics and Psychotherapy, Medical Faculty, RWTH Aachen University, Aachen, Germany; ^6^Department of Clinical and Experimental Medicine, University of Messina, Messina, Italy; ^7^Center for Studies and Research in Cognitive Neuroscience, Department of Psychology “Renzo Canestrari,” Cesena Campus, Alma Mater Studiorum Università di Bologna, Cesena, Italy; ^8^Neuropsychology and Cognitive Neurosciences Research Center (CINPSI Neurocog), Universidad Católica del Maule, Talca, Chile

**Keywords:** academic assessment, time of the day, midday, ego depletion, circadian rhythms, Gaussian adaptation

## Abstract

The influence of timing on decision-making processes has garnered significant attention across various domains, yet its impact on academic assessment remains under investigated. While previous research has suggested time-of-day effects on judicial decisions, methodological limitations have restricted the generalizability of these findings. Here, we present a comprehensive analysis of 104.552 oral exams conducted at an Italian university, revealing a robust relationship between exam timing and academic outcomes. Our results demonstrate a Gaussian distribution of passing rates throughout the day, with a significant peak at midday. This pattern persists after controlling for exam difficulty and other potential confounding factors, suggesting an intrinsic time-dependent bias in the evaluation process. Our findings not only corroborate previous research on the influence of timing on decision-making but also extend it to the realm of academic assessment. These results have profound implications for educational policy and practice, highlighting the need for strategic exam scheduling to optimize student performance and ensure equitable evaluation.

## Introduction

The timing of critical decisions can have far-reaching consequences, yet our understanding of how time of day influences evaluation processes remains limited. Human life is largely structured around scheduling—from daily activity to long-term plans—and this ability to plan and schedule offers significant advantages for resource management, rest, and social interaction. However, scheduling does not always improve efficiency, nor does it necessarily promote overall performance. Excessive scheduling can limit flexibility, reduce spontaneity, and cause dependency on routines. Importantly, evidence suggests that scheduling can cause biases in decisional processes. A seminal study by Danziger et al. (2011) revealed that the timing of judicial decisions was significantly affected by the structure of the court's schedule.

Judges were more likely to render favorable decisions at the start of a court session or immediately following meal breaks. As the session progressed, the likelihood of a favorable decision decreased, suggesting that factors like mental fatigue or ego depletion could influence the decision of judges. According to the ego depletion theory (Muraven and Baumeister, 2000), that the demands of repeated judgments could deplete executive functions and mental resources, potentially leading to less favorable outcomes later in the day. However, critiques from Weinshall-Margel and Shapard (2011) suggest that case scheduling – rather than purely time-of-day effects—might also have influenced the results, as certain cases were more likely to be heard at specific times of the day (i.e., unaccompanied prisoners usually have appointments later during the day and are less likely to be granted than prisoners accompanied by attorneys). According to Weinshall-Margel and Shapard (2011), this suggests that the reported results might have been influenced by the predetermined order (i.e., the predetermined sequence of cases) intermingled with the specific characteristics of the cases themselves, rather than solely reflecting time of day-dependent factors, such as the decision makers' fatigue or the timing of breaks.

To address previous limitations, we conducted archival research using the Esse3 multifunction academic platform of the Italian academic system. This platform is a comprehensive management system that provides students and professors with a secure area to manage exam registrations, grades, participation in university initiatives, and more. The Esse3 platform allowed us to access detailed information about the date, time, and outcomes (passed, failed) of all scheduled exams at the university. For privacy reasons, no access to individual scores was granted.

Our study focused on exams conducted from October 2018 to February 2020, specifically to avoid the period affected by the COVID-19 pandemic and the subsequent use of online platforms for examinations. This timeframe enabled us to analyze the probability of favorable assessments, i.e., passing rates, in relation to the timing of the exams (specifically, the hour at which the exam began).

Using the ESSE3 platform allowed us to explore differences in exam outcomes based on the scheduling of exams, under conditions of random exam assignment. Moreover, our study investigated whether the timing of exam sessions influenced decision-making related to performance and merit—areas that are inherently more subjective than judicial decisions, which are based on the interpretation and application of law and tend to be more rigid due to their deep roots in legal frameworks and precedents.

## Methods

Data were extracted from the Esse3 multifunction academic platform of the Italian academic system. These data comprised a total of 104.552 (exams) assessments provided by 680 examiners, covering 1.243 courses. The dataset included records collected from October 2018 to February 2020, involving 19.116 students enrolled in 78 bachelor's and master's programs, as well as post-graduate specializations (see Table 1 for details of exams/assessments across different programs). We did not consider subsequent periods, as exams during that time were conducted via online platforms due to the COVID-19 pandemics.

**Table 1:** Number of assessments and respective percentages per type of degree course.

**Type of the degree course**	**Exams**	**%**
Degree course (3 years)	63.417	60.7
Master's degree (2 years)	15.013	14.4
Master's degree (5 years single–cycle)	24.135	23.1
Master's degree (6 years single–cycle)	1.987	1.9
Total	104.552	100.0

The extracted data included information about the type of course, the start time of the assessment session (ranging from 8 a.m. to 4 p.m.), the modality of the assessment (in person), the type of assessment (oral), and the respective outcome (passed or failed coded with 1 for passed and 0 for failed). We excluded written exams, as those assessments were conducted primarily at home and did not allow for control over the timing variable.

For privacy reasons, demographic data (e.g., age, sex, country of origin) were not provided. A typical oral exam session begins with verifying the identity of the student. There is no standardized method for conducting the examination; the assessor may start the session by asking the student to select a topic from the general course program, or by choosing a topic from those covered in the course. The duration of the exam and the number of questions also vary depending on preferences of the assessors and their need to gauge the student's level of preparation. The study was approved by the Local Ethics Committee (Protocol Number: COSPECS_08_2022). The ethics committee waived the requirement for consent, as the study involved the analysis of already collected and anonymized data. All methods were performed in accordance with the relevant guidelines and regulations. Informed consent is not required due to the retrospective nature of the study.

### Data analysis

Prior to analysis, we normalized all evaluations based on the University's educational credits (CFU – “*crediti formativi universitari*” in the Italian University System) associated with each exam. CFUs quantify the workload required to complete a course or a set of courses, encompassing lectures, seminars, practical work, and individual study. Typically, one CFU is equivalent to 25 h of work. This approach allows to establish an equivalent difficulty level for exams taken at different times, thereby avoiding potential biases caused by varying degrees of difficulty associated with each exam (*i*). The weighting *W*_*i*_ of the data was calculated, for a student *i*, using the following formula:


(1) $$\begin{array}{l} W_{i}=CFU_{i}\frac{\overset{N}{\underset{i=1}{\sum }}CFU_{i}}{\overset{N}{\underset{i=1}{\sum }}Exams_{i}} \end{array}$$

with *i* = 1…..*N*.

So, the overall passing rate is calculated:


(2) $$\begin{array}{l} Passing\;rate=\frac{\overset{N}{\underset{i=1}{\sum }}Passed_{i}}{\overset{N}{\underset{i=1}{\sum }}Exams_{i}}W_{i} \end{array}$$

where *Passed*_*i*_ is equal to 1 if the exam is passed and equal to 0 if the exam is failed. A one-Way ANOVA with passing rate as dependent variable and hour of the day as between subjects factor (9 levels: from 8 a.m. to 4 p.m.) was carried out, and in case of significant results followed by *post hoc* Bonferroni-corrected Student's *t*-tests.

To explore the interaction between the 2 variables—hour of the day and passing rate – we also applied a non-parametric technique known as Chi-Square Automatic Interaction Detection (CHAID) (Kass, 1980). CHAID is a decision tree algorithm used for predictive modeling and classification. In this contest, CHAID aims to create a tree structure that predicts the target variable (Passing rate) based on the values of predictor variables (hour of the day). CHAID uses *p*-values with a Bonferroni correction as splitting criteria. The logic of testing and formulating conclusions is identical to the traditional procedure for statistical hypothesis testing, with a software algorithm support allowing for rapid computation of multiple tests and the implementation of a heuristic approach for determining the best partition of the observed data set (decision tree diagram). The total number of exams in the CHAID procedure is slightly different from the total number of exams in the sample because the data are weighted, as explained above. The terminal nodes represent the final predicted outcome.

## Results

**Figure 1:**
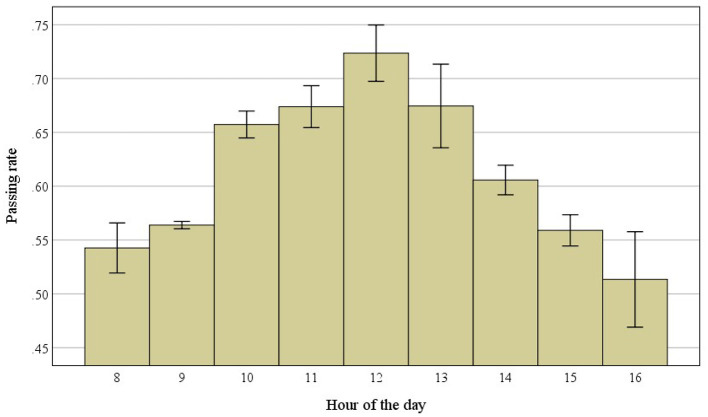
Observed distribution of the passing rate by hour. Vertical bars indicate standard error of means.

Considering the full sample, we observed 60.065 passed out of a total of 104.552 exams, resulting in an overall passing rate of 0.574 (57%). The analysis of the influence of the hour of the day on the assessment outcomes revealed that passing rates follow a Gaussian distribution (see Table 2 and Figure 1). The Gaussian distribution of the collected data was evaluated by examining the fit measures suggested in Faraway et al. (2019) and Table 2.

**Table 2:** Fit statistics for the relation of the observed distribution to a Gaussian distribution.

**Test**	**Statistic**	***p***
Kolmogorov–Smirnov	0.218	0.785
Cramér–von Mises	0.070	0.764
Anderson–Darling	0.397	0.848
Shapiro–Wilk	0.932	0.498

The One-Way ANOVA provided significant results (Table 3), with a significant main effect of the factor “hour of the day” (Table 3).

**Table 3:** One–way ANOVA test.

**Source of variability**	**Sum of squares**	***Df***	**Mean Square**	***F***	**Sig**.
Between Groups	113.39	8	14.174	58.254	< 0.001
Within Groups	25,436.15	104,543	0.243		
Total	25,549.54	104,551			

Partial eta squared (η^2^) indicates a medium effect size (η^2^ = 0.34).

As shown by the *post hoc* tests, the curve peaked at 12.00 p.m., with no significant differences between 11.00 a.m., 12.00 p.m. and 13.00 (*p* > 0.05, see Tables 4, 5 and Figure 1). Conversely, lower passing rates were found in the early morning and late afternoon, with no difference at 8.00 a.m., 9.00 a.m., 15.00, and 16.00 o'clock (*p* > 0.05, see Tables 4, 5). Finally, an intermediate passing rate was found at 10.00 a.m., which did not differ significantly from the rate at 11.00 a.m. (*p* > 0.05, see Tables 4, 5).

**Table 4:** Passing rates by hour of the day: this table shows the number of examinations conducted, along with respective standard errors (S.E.) and standard deviations (S.D.).

**Hour of the day**	Passing rate^*^	**S.E**.	**S.D**.	**Exams**
8	0.543	0.012	0.498	1,839
9	0.564	0.002	0.496	82,618
10	0.657	0.006	0.475	5,769
11	0.674	0.001	0.469	2,310
12	0.724	0.013	0.447	1,167
13	0.674	0.019	0.469	583
14	0.606	0.007	0.489	5,067
15	0.559	0.007	0.497	4,689
16	0.513	0.022	0.500	509
Total	0.574	0.002	0.494	104,552

* Passing rates are weighted according to formula (1).

**Table 5:** *Post hoc* tests with Bonferroni correction.

**(I) Hour of the day**		**Mean difference (I–J)**	**Std. error**	**Sig**.	**95% Confidence interval**	
					**Lower bound**	**Upper bound**
8	9	−0.021	0.012	1.000	−0.06	0.02
	10	−0.115^*^	0.013	< 0.001	−0.16	−0.07
	11	−0.131^*^	0.015	< 0.001	−0.18	−0.08
	12	−0.181^*^	0.018	< 0.001	−0.24	−0.12
	13	−0.132^*^	0.023	< 0.001	−0.21	−0.06
	14	−0.063^*^	0.013	< 0.001	−0.11	−0.02
	15	−0.016	0.014	1.000	−0.06	0.03
	16	0.029	0.025	1.000	−0.05	0.11
9	8	0.021	0.012	1.000	−0.02	0.06
	10	−0.0093^*^	0.007	< 0.001	−0.11	−0.07
	11	−0.0110^*^	0.010	< 0.001	−0.14	−0.08
	12	−0.0160^*^	0.015	< 0.001	−0.21	−0.11
	13	−0.0111^*^	0.020	< 0.001	−0.18	−0.05
	14	−0.0042^*^	0.007	< 0.001	−0.06	−0.02
	15	0.005	0.007	1.000	−0.02	0.03
	16	0.050	0.022	0.774	−0.02	0.12
10	8	0.0115^*^	0.013	< 0.001	0.07	0.16
	9	0.093^*^	0.007	< 0.001	0.07	0.11
	11	−0.017	0.012	1.000	−0.06	0.02
	12	−0.066^*^	0.016	0.001	−0.12	−0.02
	13	−0.017	0.021	1.000	−0.09	0.05
	14	0.052^*^	0.009	< 0.001	0.02	0.08
	15	0.098^*^	0.010	< 0.001	0.07	0.13
	16	0.144^*^	0.023	< 0.001	0.07	0.22
11	8	0.131^*^	0.015	< 0.001	0.08	0.18
	9	0.110^*^	0.010	< 0.001	0.08	0.14
	10	0.017	0.012	1.000	−0.02	0.06
	12	−0.050	0.018	0.181	−0.11	0.01
	13	−0.001	0.023	1.000	−0.07	0.07
	14	0.068^*^	0.012	< 0.001	0.03	0.11
	15	0.115^*^	0.013	< 0.001	0.07	0.15
	16	0.160^*^	0.024	< 0.001	0.08	0.24
12	8	0.181^*^	0.018	< 0.001	0.12	0.24
	9	0.160^*^	0.015	< 0.001	0.11	0.21
	10	0.066^*^	0.016	0.001	0.02	0.12
	11	0.050	0.018	0.181	−0.01	0.11
	13	0.049	0.025	1.000	−0.03	0.13
	14	0.118^*^	0.016	< 0.001	0.07	0.17
	15	0.165^*^	0.016	< 0.001	0.11	0.22
	16	0.210^*^	0.026	< 0.001	0.13	0.29
13	8	0.132^*^	0.023	< 0.001	0.06	0.21
	9	0.111^*^	0.020	< 0.001	0.05	0.18
	10	0.017	0.021	1.000	−0.05	0.09
	11	0.001	0.023	1.000	−0.07	0.07
	12	−0.049	0.025	1.000	−0.13	0.03
	14	0.069	0.022	0.052	0.00	0.14
	15	0.116^*^	0.022	< 0.001	0.05	0.18
	16	0.161^*^	0.030	< 0.001	0.07	0.26
14	8	0.063^*^	0.013	< 0.001	0.02	0.11
	9	0.042^*^	0.007	< 0.001	0.02	0.06
	10	−0.052^*^	0.009	< 0.001	−0.08	−0.02
	11	−0.068^*^	0.012	< 0.001	−0.11	−0.03
	12	−0.118^*^	0.016	< 0.001	−0.17	−0.07
	13	−0.069	0.022	0.052	−0.14	0.00
	15	0.047^*^	0.010	< 0.001	0.01	0.08
	16	0.092^*^	0.023	0.002	0.02	0.17
15	8	0.016	0.014	1.000	−0.03	0.06
	9	−0.005	0.007	1.000	−0.03	0.02
	10	−0.098^*^	0.010	< 0.001	−0.13	−0.07
	11	−0.115^*^	0.013	< 0.001	−0.15	−0.07
	12	−0.165^*^	0.016	< 0.001	−0.22	−0.11
	13	−0.116^*^	0.022	< 0.001	−0.18	−0.05
	14	−0.047^*^	0.010	< 0.001	−0.08	−0.01
	16	0.046	0.023	1.000	−0.03	0.12
16	8	−0.029	0.025	1.000	−0.11	0.05
	9	−0.050	0.022	0.774	−0.12	0.02
	10	−0.144^*^	0.023	< 0.001	−0.22	−0.07
	11	−0.160^*^	0.024	< 0.001	−0.24	−0.08
	12	−0.210^*^	0.026	< 0.001	−0.29	−0.13
	13	−0.161^*^	0.030	< 0.001	−0.26	−0.07
	14	−0.092^*^	0.023	0.002	−0.17	−0.02
	15	−0.046	0.023	1.000	−0.12	0.03

The Bonferroni adjustment multiplies the *p*–values by [k × (k−1)]/2[k \times (k−1)]/2[k × (k−1)]/2 (the number of pairwise comparisons), where k is the number of groups. ^*^The mean difference is significant at the 0.05 level.

**Figure 2:**
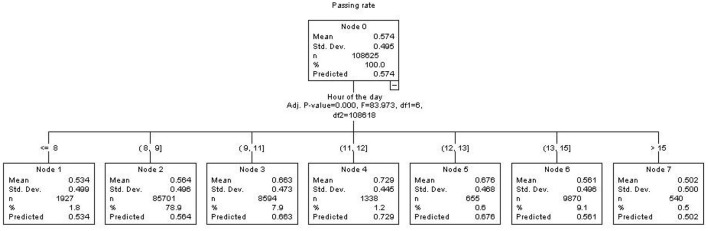
Decision tree diagram obtained by the CHAID. The terminal nodes represent the optimal partitioning of the initial data set (Node 0). The “mean” term in the terminal node indicates the pass rate.

Also the CHAID procedure provided significant results [*F*_(6, 108618)_ = 83.97, *p* < 0.001]. The CHAID output identified the best partition of the initial data set (Node 0) for 7 terminal nodes, merging the times of 10 with 11 and 14 with 15 (Figure 2).

## General discussion

In this study, we examined the hypothesis that exam scheduling influences academic assessments, focusing specifically on the influence of different exam times on passing rate. By analyzing assessments scheduled between 8:00 a.m. to 4:00 p.m., our findings revealed a Gaussian distribution of passing rates, peaking around 12:00 p.m., with an overall higher probability of passing in the late morning and a lower probability of passing in the early morning and late afternoon. This midday peak may reflect the combined effects of circadian-driven alertness and cognitive arousal patterns across the day. Research shows that general cognitive performance follows a bell-shaped curve, typically improving from morning into midday and declining afterward, due to the natural progression of physiological arousal (Folkard, 1975; Valdez et al., 2005). The midday peak is also consistent with earlier evidence that appetite could improve cognitive performance (Vicario et al., 2019). Moreover, the “post-lunch dip”—a well-documented decline in alertness and performance occurring in the early afternoon—may explain the decline observed after the midday peak (Monk, 2005). This pattern aligns with findings in chronobiology suggesting that cognitive efficiency tends to be optimal in the late morning for most individuals, especially under standardized scheduling constraints (Schmidt et al., 2007). Thus, the Gaussian-shaped distribution we observe is consistent with known circadian and homeostatic/physiological influences on cognition and performance.

Overall, these results suggest that academic evaluations vary throughout the day, likely influenced by multiple factors affecting both students and assessors. This finding aligns with previous research (e.g., Danziger et al., 2011; Vicario et al., 2018), which suggested that decision-making may vary according to the physiological state of the decision-maker. Similarly, our findings demonstrate that the context of academic assessments may be influenced by analogous factors.

## Theoretical implications

The nature of the available data does not allow us to directly determine which factors are responsible for the observed results. These outcomes may arise from a complex interplay of assessor decision-making, student performance, or both. Factors not explicitly investigated in the current work such as the circadian rhythms of students and assessors, their overall tiredness (potentially influenced by sleep quality), the number of breaks taken by assessors during the exams, and other factors, such as diurnal fluctuations of mood, may have all contributed to passing rates observed in the current study. However, the specific role and impact of these (and other potential variables) remains uncertain, warranting systematic future research on the topic.

The lower passing rates observed in the early morning might be attributable to both students and assessor factors, particularly those related to circadian rhythms and chronotype. On the student side, performance may be influenced by the natural circadian regulation of energy levels, alertness, and brain physiology (Kanarskii et al., 2022; Salehinejad et al., 2021, 2022). Studies have shown that individuals with an evening chronotype often experience reduced cognitive performance in the morning compared to later in the day (Barclay and Myachykov, 2017; Zou et al., 2022; Salehinejad et al., 2021). Given that adolescents and young adults—such as university students—are more likely to have an evening chronotype, often persisting until the age of 30 (Karan et al., 2021; West et al., 2024), this misalignment between biological rhythms and early exam scheduling could negatively affect cognitive functioning and thus explain lower passing rates during morning exams.

Equally, assessor-related factors may play a critical role in shaping outcomes. The decision-making processes of assessors could also be influenced by their own chronotype and energy levels throughout the day. For instance, assessors who prefer morning hours—common in individuals aged 40 to 60 (Fischer et al., 2017), which matches the typical age range of assessors in our sample—may exhibit greater alertness and cognitive sharpness in the early hours. This alignment could lead to more stringent grading, particularly if assessors are more sensitive to student performance shortcomings during their own peak cognitive times. Conversely, during later parts of the day, assessors may experience cognitive fatigue or ego depletion, potentially lowering their evaluation strictness. Thus, the interplay between student and assessor chronotypes and their alignment or misalignment with assessment timing may jointly contribute to the observed performance patterns, underscoring the importance of considering both perspectives in future research and scheduling policies.

## Practical implications

The concept of ego depletion (Muraven et al., 2019), which proposes that exerting self-control can deplete mental resources, offers an additional lens through which to understand the decline in passing rates observed in the afternoon. Considering that mental fatigue tends to increase progressively throughout the day due to daily activities (e.g., Song et al., 2019), and that this effect is accelerated under stress, such as during a forthcoming exam session (Oaten and Cheng, 2005), it is plausible that the progressive reduction of the passing rates in the afternoon is linked to this specific kind of fatigue likely affecting both students and assessors.

The demands of managing exam stress requires self-control to maintain focus, manage time, and resist distractions. The progressive decline in passing rates observed in the afternoon may be due to ego depletion, as students' and assessors' cognitive resources become fatigued by the examination stress, which is known to impair self-control (Oaten and Cheng, 2005), ultimately leading to reduced passing rates. Specifically, the growing rigidity or reduced flexibility associated with cognitive resource depletion (Muraven, 2012; Vohs et al., 2008) may result in a higher rejection bias in assessors, consistent with the findings of Danziger et al. (2011), suggesting that judges in a state of ego depletion were more likely to make decisions that were less favorable to defendants. Moreover, diminished self-control in students can adversely affect their concentration, directly impacting their performance (Muraven et al., 2019), reinforcing the notion that self-discipline, a key aspect of self-control, serves as a better predictor of academic success than traditional measures of intelligence (Duckworth and Seligman, 2005). The peak in passing rates around midday may reflect the optimal balance between chronotype alignment and mental depletion, according to the explanations provided above. Overall, these results support previous evidence (e.g., Danziger et al., 2011) that decisional processes can be dramatically influenced by the time of day when evaluations take place. This suggests that, to optimize academic performance and ensure fairer assessments, educational institutions should consider scheduling important exams and assessments during the late morning to early afternoon, as this time frame covers the most favorable period of the day for passing rates according to our data.

## Limitations and future research

Our results offer valuable insights into optimizing outcomes across various domains by considering the effects of time-of-day on decision-making of assessors and student performance. Research on how diurnal timing influences decision-making processes and performance could inform different fields, including workplaces, healthcare, sports competition, consumer behavior, and public policy. Yet, future research should further explore the underlying mechanisms of time-of-day effects considering not only chronotype and mental depletion but also factors like subjective stress and emotion. A more comprehensive investigation into potential confounding variables is warranted. These may include sleep quality and duration, emotional state, prior cognitive load, environmental conditions (e.g., lighting, noise, and temperature), and unmeasured demographic factors such as age, socioeconomic background, and prior achievement levels. Accounting for these factors is essential to avoid over-attributing performance effects solely to time-of-day variations.

Equally important is the need for future research to systematically address the role of circadian rhythms and ego depletion. Circadian rhythms—individual physiological cycles that influence alertness and performance—can vary widely across individuals, especially between morning and evening chronotypes. Aligning assessment timing with individual circadian preferences could improve fairness and accuracy in performance evaluations. Moreover, ego depletion, could have disproportionately affect assessors across the day, leading to inconsistency in grading or decision-making. Integrating physiological and psychological measures of depletion and alertness (e.g., cortisol levels, subjective fatigue, or reaction time tasks) could help clarify how these factors interact with diurnal timing to impact both assessor and student outcomes. This might necessitate relatively broad assessments, enabling to identify potential strategies to increase fairness of assessments outside of the optimal time window. Furthermore, the generalizability of our findings to other institutions and populations should be approached with caution. As the data originate from a specific academic context, differences in institutional structures and/or respective university polices, cultural norms, scheduling practices, and student demographics may limit the applicability of our results elsewhere. Future studies across diverse countries/educational systems and cultural settings are necessary to determine the extent to which these time-of-day effects hold more broadly. Including more varied populations would help clarify whether the observed patterns are universal or context-dependent. Finally, as a further limitation, we acknowledge that although course difficulty was controlled for, exam difficulty may still have varied across different times of the day.

These strategies may involve advising students to adjust to exam schedules, prioritize sufficient sleep, and similar recommendations for assessors. If chronotype is a key factor, exam times could be adjusted accordingly. To mitigate the impact of ego depletion among assessors, reducing the number of exams conducted per session and incorporating more frequent breaks could prove beneficial. Furthermore, delaying the start of exams in line with the evidence showing that a 1-h delay in school start times improves attention, performance, and reduces impulsivity (Lufi et al., 2011) could further improve outcomes. Impulsivity, which is known to affect self-control (Lucifora et al., 2021), tends to escalate under conditions of ego depletion (Vohs and Faber, 2007), potentially diminishing passing rates.

While the exact mechanisms underlying our findings remain largely speculative, the proposed candidates provide a robust foundation for proposing behavioral and scheduling changes that could enhance assessment fairness. Thus, our results offer actionable insights with direct applications, while underscoring the necessity for further mechanistic research in this domain.

## Data Availability

The data analyzed in this study is subject to the following licenses/restrictions: The owner of the data is the University of Messina. Requests to access these datasets should be directed to massimo.mucciardi@unime.it.
