# Correction: Timing matters! Academic assessment changes throughout the day

**DOI:** 10.3389/fpsyg.2025.1678551

**Published:** 2025-08-13

**Authors:** Carmelo M. Vicario, Michael A. Nitsche, Chiara Lucifora, Pietro Perconti, Mohammad A. Salehinejad, Francesco Tomaiuolo, Simona Massimino, Alessio Avenanti, Massimo Mucciardi

**Affiliations:** ^1^Dipartimento di Scienze Cognitive, Psicologiche, Pedagogiche e degli studi culturali, Università di Messina, Messina, Italy; ^2^Department of Psychology and Neurosciences, Leibniz Research Centre for Working Environment and Human Factors (IfADo), Dortmund, Germany; ^3^Dipartimento di Filosofia e Comunicazione, Università di Bologna, Bologna, Italy; ^4^School of Cognitive Sciences, Institute for Research in Fundamental Sciences (IPM), Tehran, Iran; ^5^Department of Child and Adolescent Psychiatry, Psychosomatics and Psychotherapy, Medical Faculty, RWTH Aachen University, Aachen, Germany; ^6^Department of Clinical and Experimental Medicine, University of Messina, Messina, Italy; ^7^Center for Studies and Research in Cognitive Neuroscience, Department of Psychology “Renzo Canestrari,” Cesena Campus, Alma Mater Studiorum Università di Bologna, Cesena, Italy; ^8^Neuropsychology and Cognitive Neurosciences Research Center (CINPSI Neurocog), Universidad Católica del Maule, Talca, Chile

**Keywords:** academic assessment, time of the day, midday, ego depletion, circadian rhythms, Gaussian adaptation

Affiliations 4 “School of Cognitive Sciences, Institute for Research in Fundamental Sciences (IPM), Tehran, Iran” and 5 “Department of Child and Adolescent Psychiatry, Psychosomatics and Psychotherapy, Medical Faculty, RWTH Aachen University, Aachen, Germany” were omitted for author Mohammad A. Salehienjad. These affiliations have now been added for author Mohammad A. Salehienjad.

Affiliation 8 “Neuropsychology and Cognitive Neurosciences Research Center (CINPSI Neurocog), Universidad Católica del Maule, Talca, Chile” was omitted for author Alessio Avenanti. This affiliation has now been added for author Alessio Avenanti.

The original version of this article has been updated.

